# Increasing hospitalization and death possibly due to Clostridium difficile diarrheal disease.

**DOI:** 10.3201/eid0404.980412

**Published:** 1998

**Authors:** F Frost, G F Craun, R L Calderon

**Affiliations:** Southwest Center for Managed Care Research, Albuquerque, New Mexico 87108, USA. ffrost@lrri.org

## Abstract

This study calculated yearly estimated national hospital discharge (1985 to 1994) and age-adjusted death rates (1980 to 1992) due to bacterial, viral, protozoal, and ill-defined enteric pathogens. Infant and young child hospitalization (but not death) rates in each category increased more than 50% during 1990 to 1994. Age-adjusted death and hospitalization rates due to enteric bacterial infections and hospitalizations due to enteric viral infections have increased since 1988. The increases in hospitalization and death rates from enteric bacterial infections were due to a more than eightfold increase in rates for specified enteric bacterial infections that were uncoded during this period (ICD9 00849). To identify bacterial agents responsible for most of these infections, hospital discharges and outpatient claims (coded with more detail after 1992) were examined for New Mexico's Lovelace Health Systems for 1993 to 1996. Of diseases due to uncoded enteric pathogens, 73% were due to Clostridium difficile infection. Also, 88% of Washington State death certificates (1985 to 1996) coded to unspecified enteric pathogen infections (ICD0084) listed C. difficile infection.


### Increasing Hospitalization and Death Possibly Due to *Clostridium difficile* Diarrheal Disease

**Floyd Frost,* Gunther F. Craun,† and Rebecca L. Calderon‡**
*Southwest Center for Managed Care Research, Albuquerque, New Mexico, USA; †Gunther F. Craun and Associates, Staunton, Virginia, USA; and ‡U.S. Environmental Protection Agency, Research Triangle Park, North Carolina, USA

This study calculated yearly estimated national hospital discharge (1985 to 1994) and age-adjusted death rates (1980 to 1992) due to bacterial, viral, protozoal, and ill-defined enteric pathogens. Infant and young child hospitalization (but not death) rates in each category increased more than 50% during 1990 to 1994. Age-adjusted death and hospitalization rates due to enteric bacterial infections and hospitalizations due to enteric viral infections have increased since 1988. The increases in hospitalization and death rates from enteric bacterial infections were due to a more than eightfold increase in rates for specified enteric bacterial infections that were uncoded during this period (ICD9 00849). To identify bacterial agents responsible for most of these infections, hospital discharges and outpatient claims (coded with more detail after 1992) were examined for New Mexico's Lovelace Health Systems for 1993 to 1996. Of diseases due to uncoded enteric pathogens, 73% were due to *Clostridium difficile* infection. Also, 88% of Washington State death certificates (1985 to 1996) coded to unspecified enteric pathogen infections (ICD0084) listed *C. difficile* infection.

Infectious diarrhea remains a major cause of death worldwide (1). In the United States, enteric pathogens are estimated to cause 25 to 99 million episodes of diarrhea and vomiting each year, resulting in 2.2 million physician visits (2). U.S. residents at highest risk for severe illness or death from diarrhea are young children (3-5) and the elderly (5). Race, socioeconomic status, and residence in a nursing home are also risk factors for death due to diarrhea (1-6).

Increasing concern over waterborne transmission of enteric pathogens (7-10) prompted this study. Since hospitalization or death from infectious diarrhea is uncommon, state- or hospital-specific studies are unlikely to include enough cases to accurately estimate the incidence or describe the temporal trends in hospitalization or death rates. Therefore, this study focused on national hospitalization and death data to determine if the incidence of hospitalizations or deaths due to infectious diarrhea has changed during the past decade and, if so, to identify specific pathogens responsible for the changes.

Address for correspondence: Floyd Frost, Southwest Center for Managed Care Research, 2425 Ridgecrest Drive, S.E., Albuquerque, NM 87108, USA; fax: 505-262-7598; e-mail: ffrost@lrri.org.

### Data Sources

Computerized data on U.S. death rates (1980 to 1992), which included coded underlying cause of death, and National Hospital Discharge Survey (NHDS) data (1985 to 1994) were obtained from the National Center for Health Statistics (NCHS). NHDS data came from yearly surveys of hospital discharges conducted by NCHS through a multistage sampling scheme (11). Yearly national estimates of discharges by diagnosis, age, and gender were obtained by using multipliers provided by NCHS. Yearly midyear Bureau of the Census population estimates were used for calculating national death and hospitalization rates.

### Deaths Due to Diarrhea, 1980–1992

The number of diarrhea deaths in 1980 to 1992 was determined by using the coded underlying causes of death. Deaths are coded to only four digits of the International Classification of Disease 9th revision (ICD9). Causes of death were grouped into four categories according to type of pathogen: bacterial, parasitic, viral, and ill-defined (Table 1).

### Hospitalizations Due to Diarrhea, 1985–1994

Hospitalizations due to diarrhea (coded to five digits of the ICD9) were ascertained for each year from 1985 to 1994 by selecting the same groups of ICD9 codes from the first seven discharge diagnoses recorded for each hospital discharge. For the few hospital discharges with multiple infectious diarrhea codes, priority was given to the code that appeared first.

### Hospitalization and Death Rates Adjusted by Age

Age-adjusted hospitalization and death rates for each year, standardized to the 1990 U.S. population, were calculated for each of the four ICD9 enteric pathogen categories (bacterial, parasitic, viral, and ill-defined). A ratio of deaths to hospitalizations for each category was calculated by dividing the number of deaths caused by pathogens in each category by the number of hospital discharges with the first infectious enteric disease discharge diagnosis included in that category.

### Hospitalizations and Deaths–Bacterial Causes

Hospitalizations and deaths due to bacterial causes (including cholera, typhoid and paratyphoid fever, other salmonella infections, shigellosis, other food poisoning bacteria, and infections due to other specified bacteria) were also analyzed (Table 1). Age-adjusted death rates (each year) from "other specified enteric bacteria" (ICD9 0084) and age-adjusted hospitalization rates (each year) due to "uncoded but specified enteric bacteria" (ICD9 00849) were calculated. Additional cause codes were added in 1992 (ICD9 00843-00847 for hospital ICD coding, including 00845 for *Clostridium difficile* (Table 1). However, these codes were not yet incorporated for this analysis of national hospital discharge data. During this time, cause of death was only coded to the fourth ICD9 digit.

Table 1. International Classification of Disease (ICD9) Categories

| | |
|---|---|
| Bacterial | |
| 001[a] | Cholera |
| 002[a] | Typhoid and paratyphoid fevers |
| 003[a] | Other salmonella infections |
| 004[a] | Shigellosis |
| 005[a] | Other food poisoning bacterial |
| 008[a] | Intestinal infections due to other organisms |
| 0080 | *Escherichia coli* |
| 0081 | Arizona group of paracolon bacilli |
| 0082 | *Aerobacter aerogenes* (Enterobacter) |
| 0083 | Proteus |
| 0084 | Other specified bacteria |
| 0085 | Bacterial enteritis (unspecified) |
| 041[a] | Bacterial infection in conditions classified elsewhere and of unspecified site |
| For hospital coding | |
| 00841 | Staphylococcus |
| 00842 | Pseudomonas |
| 00849 | Other |
| After 1992 | |
| 00843 | Campylobacter |
| 00844 | *Yersinia enterocolitica* |
| 00845 | *Clostridium difficile* |
| 00846 | Other anaerobes |
| 00847 | Other gram-negative bacteria |
| Viral | |
| 074[a] | Specified disease due to coxsackie virus |
| 045[a] | Acute poliomyelitis |
| 047[a] | Meningitis due to enterovirus |
| 048 | Other enterovirus diseases of central nervous system |
| 0086 | Enteritis due to specified virus |
| 0088 | Other viral organism not elsewhere classified |
| 0700[a] | Viral hepatitis A with hepatic coma |
| 0701[a] | Viral hepatitis A without mention of hepatic coma |
| Parasitic | |
| 006[a] | Amebiasis |
| 007[a] | Other protozoal intestinal diseases |
| 127[a] | Other intestinal helminthiases |
| 128 | Other and unspecified helminthiases |
| 129[a] | Intestinal parasitism, unspecified |
| Other | |
| 009[a] | Ill-defined intestinal infections |

[a] Diarrhea codes selected for study.

### Deaths (1985–1996) and Hospitalizations (1993–1996)

To make use of enhanced ICD9 coding available after 1992, inpatient health-care records for 1993 to 1996 from the Lovelace Health Systems in Albuquerque, New Mexico, were reviewed to identify bacterial pathogens responsible for most hospitalizations that would have been coded (before 1992) to specified but uncoded bacterial pathogens. Since death records are only coded to the fourth digit, Washington State deaths (with any multiple cause of death code of ICD9 0084) occurring between 1985 and 1996 were also reviewed to identify the specific pathogen that resulted in this ICD9 code assignment.

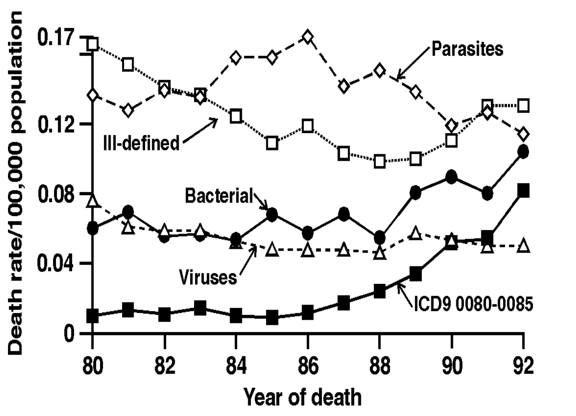

Figure 1. Age-adjusted death rates per 100,000 population by grouped underlying cause of death for selected enteric pathogens, United States 1980-92. (Standardized to the 1970 U.S. population).

### Findings

### Death Rates

Age-adjusted death rates for protozoal, viral, and other causes remained relatively stable from 1980 to 1992 (Figure 1), but age-adjusted death rates for bacterial causes increased from 0.060 per 100,000 population in 1980 to 0.104 per 100,000 in 1994, more than 60% ($p < 0.00001$) (Figure 1). To ascertain the specific bacterial agents responsible for the increase, deaths were initially categorized by each three- and four-digit ICD9 code included in the bacterial category. The age-adjusted death rate due to other specified bacterial pathogens (ICD9 0080-0084) increased from 0.0102 per 100,000 population to 0.0821 per 100,000 population ($p < 0.000001$), more than eightfold (Table 2). This increase accounted for the overall increase in the age-adjusted death rate for deaths due to enteric bacterial pathogens, as the age-adjusted death rate for other enteric bacterial causes remained stable or declined.

Most of the increase in the age-adjusted death rate for ICD9 0080-0084 was due to a statistically significant ($p < 0.00001$) increase in the death rate of persons age 45 years and older

Table 2. Age-adjusted death rates for bacterial causes of diarrhea

| | ICD9 0080-0084[a] | | | | Remainder of bacterial causes[b] | | | |
|---|---|---|---|---|---|---|---|---|
| Year | AARATE[c] | <45 yrs[d] | ≥45 yrs[d] | (Deaths) | AARATE[3] | <45 yrs[d] | ≥45 yrs[d] | (Deaths) |
| 1980 | 0.0102 | 0.0013 | 0.0090 | (21) | 0.0500 | 0.0133 | 0.0368 | (107) |
| 1981 | 0.0135 | 0.0035 | 0.0100 | (29) | 0.0559 | 0.0107 | 0.0453 | (120) |
| 1982 | 0.0107 | 0.0022 | 0.0085 | (23) | 0.0463 | 0.0087 | 0.0365 | (100) |
| 1983 | 0.0144 | 0.0040 | 0.0104 | (32) | 0.0426 | 0.0092 | 0.0334 | (94) |
| 1984 | 0.0101 | 0.0026 | 0.0075 | (23) | 0.0433 | 0.0068 | 0.0365 | (98) |
| 1985 | 0.0092 | 0.0016 | 0.0075 | (21) | 0.0587 | 0.0131 | 0.0456 | (135) |
| 1986 | 0.0119 | 0.0029 | 0.0090 | (28) | 0.0485 | 0.0108 | 0.0347 | (105) |
| 1987 | 0.0175 | 0.0029 | 0.0146 | (41) | 0.0506 | 0.0097 | 0.0409 | (120) |
| 1988 | 0.0241 | 0.0008 | 0.0232 | (57) | 0.0305 | 0.0071 | 0.0234 | (74) |
| 1989 | 0.0342 | 0.0016 | 0.0326 | (83) | 0.0465 | 0.0111 | 0.0354 | (114) |
| 1990 | 0.0520 | 0.0043 | 0.0478 | (129) | 0.0374 | 0.0070 | 0.0303 | (93) |
| 1991 | 0.0547 | 0.0027 | 0.0520 | (139) | 0.0254 | 0.0055 | 0.0190 | (65) |
| 1992 | 0.0821 | 0.0046 | 0.0775 | (213) | 0.0220 | 0.0049 | 0.0171 | (57) |

[a] Causes include *Escherichia coli* (0080), Arizona group (0081), *Aerobacter aerogenes* (Enterobacter) (0082), *Proteus mirabilus* and *morganii* (0083), other specified enteric bacterial infections (0084).
[b] Causes include cholera (001), typhoid and paratyphoid fever (002), other salmonella infections (003), shigellosis (004), bacterial enteritis, unspecified (0085), bacterial infection in conditions classified elsewhere (041).
[c] Age-adjusted death rates per 100,000 population, adjusted to the 1990 U.S. population.
[d] Contribution to age-adjusted death rate from persons younger than age 45 years and from persons age 45 years and older.

(Table 2). For both older and younger age groups, the increase was most apparent from 1988 to 1992. During this time, approximately 96% of U.S. deaths coded to ICD9 0080-0084 were coded to ICD9 0084, which includes other specified but uncoded enteric bacterial pathogens.

### Hospitalization Rates

The estimated number of hospitalizations in the United States coded to infectious enteric agents increased from 131,252 to 337,178 from 1985 to 1994, a 2.5-fold increase. Age-adjusted hospitalization rates for viral causes increased more than twofold, whereas rates for bacterial causes increased more than fourfold (Figure 2). Hospitalization rates for protozoal and ill-defined causes remained stable or fluctuated from year to year (Figure 2). In 1985, bacterial causes accounted for 21% of all hospitalizations for infectious enteric agents, while in 1994, they accounted for 38% (Figure 2). A large increase was apparent in the age-adjusted hospitalization rate for other specified bacterial pathogens (ICD9 00800-00849) (Figure 2).

The increased age-adjusted rate of hospital discharge coded to "other specified bacterial pathogen" infections (ICD9 0080-00849) was statistically significant ($p < 0.00001$) (Table 3). The age-adjusted hospitalization rate for bacterial causes, other than ICD9 0080-00849, remained stable (Table 3). As with deaths, the increase in hospitalizations for bacterial causes

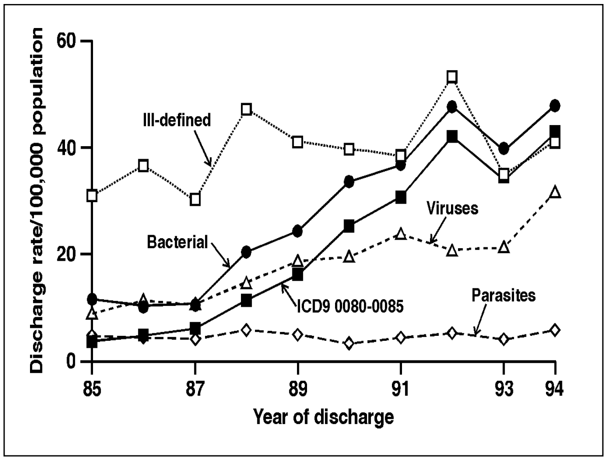

Figure 2. Age-adjusted hospital discharge rates per 100,000 population by grouped discharge diagnosis for selected enteric pathogens, United States 1985-94. (Standardized to the 1970 U.S. population). Discharges were included if a selected enteric pathogen was among the first seven discharge diagnoses. The pathogen group was assigned according to the first pathogen listed.

appears to have begun in 1988, but unlike the increase in death rates, hospitalization rates increased for persons both younger and older than 45 years of age ($p < 0.001$) (Table 3). For discharges coded to ICD9 0080-00849, 88% were coded to ICD9 00849, which includes "other uncoded but specified enteric bacterial pathogens." Discharge rates due to bacterial causes of diarrhea increased in each age group, with

Table 3. Age-adjusted hospitalization rates for bacterial causes of diarrhea

| | ICD9 00800-00849[a] | | | | Remainder of bacterial causes[b] | | | |
|---|---|---|---|---|---|---|---|---|
| Year | AARATE[c] | <45 yrs[d] | ≥ 45 yrs[d] | (Discharges) | AARATE[c] | <45 yrs[d] | ≥45 yrs[d] | (Discharges) |
| 1985 | 3.72 | 1.71 | 2.02 | (8,716) | 7.76 | 4.06 | 3.70 | (18,286) |
| 1986 | 4.79 | 2.01 | 2.74 | (11,316) | 5.56 | 2.60 | 2.96 | (13,174) |
| 1987 | 6.05 | 1.95 | 4.10 | (14,433) | 4.55 | 2.14 | 2.41 | (11,037) |
| 1988 | 11.36 | 3.20 | 8.14 | (27,492) | 9.07 | 6.04 | 3.02 | (22,219) |
| 1989 | 16.09 | 5.64 | 11.05 | (38,894) | 8.10 | 4.96 | 3.14 | (19,931) |
| 1990 | 25.23 | 6.40 | 18.82 | (62,750) | 8.28 | 4.29 | 4.00 | (21,037) |
| 1991 | 30.67 | 7.55 | 23.12 | (78,100) | 6.05 | 3.52 | 2.53 | (15,620) |
| 1992 | 41.96 | 9.71 | 32.25 | (107,874) | 5.56 | 3.15 | 2.42 | (14,568) |
| 1993 | 33.91 | 8.32 | 25.60 | (88,715) | 5.51 | 3.54 | 1.98 | (14,454) |
| 1994 | 42.66 | 10.48 | 30.18 | (113,411) | 5.22 | 3.44 | 1.78 | (13,944) |

[a]Causes include *Escherichia coli* (0080), Arizona group (0081), *Aerobacter aerogenes* (Enterobacter) (0082), *Proteus mirabilus* and *morganii* (0083), *Staphylococcus* (00841), *Pseudomonas* (00842), *Campylobacter* (00843), *Yersinia enterocolitica* (00844), *Clostridium difficile* (00845), other anaerobes (00846), other gram-negative (00847), other (00849). Codes 00843-00847 were added in 1992.

[b]Causes include cholera (001), typhoid and paratyphoid fever (002), other salmonella infections (003), shigellosis (004), bacterial enteritis, unspecified (0085), bacterial infection in conditions classified elsewhere (041).

[c]Age-adjusted discharge rates per 100,000 population, adjusted to the 1990 U.S. population.

[d]Contribution to age-adjusted discharge rate from persons younger than age 45 years and from persons age 45 years and older.

greater increases seen in children and persons more than 45 years of age (Table 4).

Infant hospitalization rates increased for bacterial, viral, protozoal, and ill-defined conditions ($p < 0.001$) (Table 4). Hospitalization rates also increased for children ages 1 to 4 years of age for bacterial, viral, and ill-defined conditions ($p < 0.001$) (Table 4); however, no increases in children's death rates were observed.

### Death-to-Discharge Ratio

The ratio of deaths to hospitalizations for protozoal and ill-defined causes of diarrhea remained stable between 1985 and 1992. However, despite the increasing incidence of enteric bacteria-caused deaths, the ratio of deaths to hospitalizations declined from 6.6 per 1,000 to 2.7 per 1,000 hospitalizations ($p < 0.001$). The ratio of hospitalizations to deaths in the viral cause category declined from 6.0 per 1,000 to 2.9 per 1,000 hospitalizations ($p < 0.001$).

### *Clostridium difficile* Infections

Additional disease codes for hospital discharge coding were added in 1992, reducing the number of discharges coded to ICD9 00849. Examination of all computerized health-care billing records (1993 to 1996) with an ICD9 of 00843-00849 from the Lovelace Hospital and Lovelace Health Care Systems in Albuquerque, New Mexico, found 94 inpatients with these codes; *C. difficile* (ICD9 00845) accounted for 73%. This suggests that *C. difficile* infection was likely to have been the most common pathogen previously coded to ICD9 00849. Eighty-six percent of patients diagnosed with *C. difficile* were younger than age 60 with 65% younger than age 40. In addition, the records of 22 (88%) of 25 Washington State deaths occurring between 1985 and 1996 with a multiple cause of death code ICD9 0084 cited *C. difficile.*

### Conclusions

Infectious diarrhea remains an uncommon cause of hospitalization and accounted for almost the same number of deaths in 1992 as in 1980. Increases in death rates for bacterial causes offset stable or declining death rates for viral, parasitic, and ill-defined causes of diarrhea. The increase in death rates for "other specified enteric bacteria" was due to increases in deaths associated with ICD9 code 0084, "uncoded but specified bacterial pathogens." Increases in

Table 4. Age-specific rates of hospitalizations (estimated discharges) by diagnostic group

| | Discharge rate/100,000 population (discharges) | |
|---|---|---|
| Age | 1985-89 | 1990-94 |
| Bacterial causes[a] | | |
| 1 | 57.5 (10,374) | 100.8 (19,536) |
| 1-4 | 7.5 (5,416) | 21.5 (16,696) |
| 5-14 | 4.6 (7,988) | 8.2 (14,988) |
| 15-24 | 7.6 (14,739) | 10.6 (19,216) |
| 25-34 | 13.0 (26,955) | 13.5 (28,583) |
| 35-44 | 10.1 (17,089) | 18.5 (36,871) |
| 45-54 | 11.1 (13,551) | 26.4 (36,152) |
| 55-64 | 18.1 (19,302) | 41.7 (43,774) |
| 65-74 | 34.9 (30,182) | 95.4 (87,902) |
| 75 | 65.9 (39,903) | 211.8 (145,965) |
| Protozoal causes[b] | | |
| 1 | 3.8 (680) | 9.1 (1,767) |
| 1-4 | 3.0 (2,157) | 2.6 (2,006) |
| 5-14 | 1.4 (2,508) | 1.3 (2,426) |
| 15-24 | 3.3 (6,337) | 1.8 (3,228) |
| 25-34 | 4.3 (8,934) | 5.6 (11,941) |
| 35-44 | 5.7 (9,723) | 5.0 (10,044) |
| 45-54 | 4.8 (5,902) | 2.8 (3,894) |
| 55-64 | 4.5 (4,786) | 3.4 (3,611) |
| 65-74 | 9.0 (7,783) | 5.0 (4,640) |
| 75 | 12.4 (7,507) | 6.6 (4,561) |
| Viral causes[c] | | |
| 1 | 167.6 (30,234) | 387.7 (75,120) |
| 1-4 | 8.9 (6,400) | 67.1 (52,021) |
| 5-14 | 8.3 (14,566) | 16.3 (29,617) |
| 15-24 | 10.4 (20,142) | 13.5 (24,577) |
| 25-34 | 15.3 (31,694) | 17.4 (36,913) |
| 35-44 | 8.8 (14,837) | 11.4 (22,707) |
| 45-54 | 6.7 (8,209) | 8.3 (11,312) |
| 55-64 | 10.6 (11,231) | 5.4 (5,658) |
| 65-74 | 10.4 (9,020) | 8.1 (7,481) |
| 75 | 18.8 (11,390) | 15.8 (10,893) |
| Ill-defined diarrheal causes[d] | | |
| 1 | 178.9 (32,272) | 283.9 (55,006) |
| 1-4 | 27.0 (19,452) | 62.8 (48,694) |
| 5-14 | 13.3 (23,301) | 17.3 (31,594) |
| 15-24 | 24.4 (47,242) | 18.2 (32,966) |
| 25-34 | 30.3 (62,701) | 21.7 (45,966) |
| 35-44 | 25.7 (43,618) | 19.4 (38,516) |
| 45-54 | 29.5 (35,972) | 28.9 (39,459) |
| 55-64 | 46.8 (49,773) | 32.6 (34,245) |
| 65-75 | 75.5 (65,336) | 50.2 (46,257) |
| 75 | 114.7 (69,476) | 93.1 (64,163) |

[a]Discharges with an enteric bacteria diagnosis (ICD9 001-005, 0080-0085, 041).
[b]Discharges with an enteric parasite diagnosis (ICD9 006-007, 127, 129).
[c]Discharges with an enteric virus diagnosis (ICD9 0086, 0088, 045, 047, 048, 0700, 0701, 074).

hospitalization rates for these "uncoded but specified bacterial pathogens" correspond temporally to the increase in death rates coded to ICD9 0084. A review of recent hospitalization records from a New Mexico health maintenance organization and death records from Washington State show that *C. difficile* was the most common pathogen in this coding group. These findings suggest that *C. difficile* was likely to have been responsible for the increase in both age-adjusted hospitalization and death rates from enteric bacterial pathogens.

Increases in death and hospitalization rates due to bacterial causes may simply indicate improved ICD9 coding for both hospitalization and death or increased diagnostic accuracy. New kits for detection of *C. difficile* toxins in stool samples may have resulted in increased *C. difficile* diagnoses. If so, the increase in enteric bacterial infections, likely due to *C. difficile,* may not be a true increase in illness from this pathogen. If this were the case, however, one might predict a corresponding reduction in hospitalizations and deaths from ill-defined diarrheal causes. Death rates for ill-defined causes of diarrhea presumed to be infectious actually rose somewhat during 1990 to 1992, and hospitalization rates have remained stable. Further studies are needed to determine if this increased age-adjusted hospitalization and death rate is due to increased *C. difficile*–associated disease and, if so, to identify risk factors for infection and disease.

Additional information about the causes of increased hospitalization and death from enteric bacterial pathogens could be provided by a review of computerized health-care delivery records. *C. difficile* can be associated with almost any antibiotic therapy, but it has been particularly associated with aminopenicillins, cephalosporins, and clindamycin, which have greater effects on the intestinal flora (12). Other factors trigger *C. difficile* toxin–associated colitis (13). Records from health maintenance organizations containing prior diagnoses and pharmaceutical treatments may provide better understanding of the risk factors.

In this study, data from one hospital system in one state and deaths occurring in one state suggest that a likely cause of the national increase in hospitalizations and deaths due to enteric bacteria may be *C. difficile* infection. It is possible that *C. difficile* is a relatively more important cause of hospitalization at Lovelace Health Systems in New Mexico and of death in Washington State than elsewhere in the United States.

The age-specific rates of hospital discharge coded to enteric bacterial, viral, and ill-defined conditions increased for children under 5 years of age. Reasons for these increases merit further inquiry. No increases in death rates from these pathogens were observed in these age groups. However, since adverse outcomes of infectious diarrhea that requires hospitalization may indicate problems with access to health care, information on the socioeconomic characteristics of the families of these children would be of interest.

Dr. Frost is an epidemiologist and the director of the Southwest Center for Managed Care Research in Albuquerque, New Mexico. His research interests include the study of waterborne diseases and the use of health-care data for promoting public health programs within managed care organizations.

### References

1. Murray CJL, Lopex AD. The global burden of disease: a comprehensive assessment of mortality and disability from diseases, injuries and risk factors in 1990 and projected to 2020. Geneva: World Health Organization; 1996.
2. Garthright WE, Archer DI, Kvenberg JE. Estimates of incidence and cost of intestinal infectious diseases in the United States. Public Health Rep 1988;103:107-15.
3. Gibson JJ, Alexander GR. Correlates of infant death from infectious diarrhea in the southeastern United States. South Med J 1985;78:26-30.
4. Mel-Shang H, Glass RI, Pinsky PF, Young-Okoh N, Sappenfield WM, Buehler JW, et al. Diarrhea deaths in American children: are they preventable? JAMA 1988;260:3281-5.
5. Lew JF, Glass RI, Gangarosa RE, Cohen IP, Bern C, Moe CL. Diarrhea deaths in the United States, 1979 through 1987. JAMA 1991;265:3280-4.
6. Cheney CP, Wong RKH. Acute infectious diarrhea. Gastrointestinal Emergencies 1993;77:1169-90.
7. Moore AC, Herwaldt BL, Craun FG, Calderon RL, Highsmith AK, Juranek DD. Surveillance for waterborne disease outbreaks - United States 1991-92. MMWR CDC Surveill Summ 1993;42(5):1-22.
8. Calderon RL, Johnson CC, Craun GF, Dufour AP, Karlin RK, Sinks T, et al. Health risks from contaminated water: do class and race matter? Toxicol Ind Health 1993;9:879-900.
9. Kramer MH, Herwaldt BL, Craun GF, Calderon RL, Juranek DD. Surveillance for waterborne-disease outbreaks—United States 1993-94. MMWR CDC Surveill Summ 1996;45:1-33.
10. Solo-Gabriele H, Neumeister S. U.S. outbreaks of cryptosporidiosis. Journal of the American Water Works Association 1996;(Sept):76-84.

11. Simmons WR. Development of the design of the NCHS hospital discharge survey. Vital Health Statistics 1970;2(39).
12. Job ML, Jacobs NF. Drug-induced *Clostridium difficile*-associated disease. Drug Saf 1997:17:37-46.
13. Caputo GM, Weitekamp MR, Bacon AE, Whitener C. *Clostridium difficile* infection: a common clinical problems for the general internist. J Gen Intern Med 1994;9(9):528-33.
